# Assessment of new HDAC inhibitors for immunotherapy of malignant pleural mesothelioma

**DOI:** 10.1186/s13148-018-0517-9

**Published:** 2018-06-18

**Authors:** Douae Bensaid, Thibaut Blondy, Sophie Deshayes, Virginie Dehame, Philippe Bertrand, Marc Grégoire, Mohammed Errami, Christophe Blanquart

**Affiliations:** 1 CRCINA, INSERM, Université d’Angers, Université de Nantes, Nantes, France; 2 Laboratory of Physiology and Physiopathology, Faculty of Sciences of Tetouan, Avenue de Sebta Mhannech II, 93002 Tetouan, BP Morocco; 3 Institut de Chimie des Milieux et Matériaux de Poitiers, UMR CNRS 7285, 4 rue Michel Brunet, TSA 521106, B27, 86073 Poitiers, France; 4 Réseau épigénétique du Cancéropôle Grand Ouest, Nantes, France; 5 CRCINA, IRS-UN, 8 Quai Moncousu, BP70721, 44007 Nantes Cedex 1, France

**Keywords:** Malignant pleural mesothelioma, HDAC inhibitors, Decitabine (5-aza-2′-deoxycytidine), Epigenetic drugs, Immunotherapy, Tumor antigen, CD8+ T-cell clone, PD-L1

## Abstract

**Background:**

Malignant pleural mesothelioma (MPM) is a very rare and highly aggressive cancer of the pleura associated in most cases with asbestos exposure. To date, no really efficient treatments are available for this pathology. Recently, it has been shown that epigenetic drugs, particularly DNA methylation or histone acetylation modulating agents, could be very efficient in terms of cytotoxicity for several types of cancer cells. We previously showed that a hypomethylating agent (decitabine) and a histone deacetylase inhibitor (HDACi) (valproic acid (VPA)) combination was immunogenic and led to the induction of an anti-tumor immune response in a mice model of mesothelioma. However, VPA is not very specific, is active at millimolar concentrations and is responsible for side effects in clinic. To improve this approach, we studied four newly synthetized HDACi, two hydroxamates (ODH and NODH) and two benzamides (ODB and NODB), in comparison with VPA and SAHA. We evaluated their toxicity on immune cells and their immunogenicity on MPM cells in combination with decitabine.

**Results:**

All the tested HDACi were toxic for immune cells at high concentrations. Combination with decitabine increased toxicity of HDACi only towards T-cell clone. A decrease in the proportion of regulatory T cells and natural killer cells was observed in particular with VPA and ODH. In MPM cells, all HDACi combinations induced NY-ESO-1 cancer testis antigen (CTA) expression and the recognition of the treated cells by a NY-ESO-1 specific T-CD8 clone. However, for MAGE-A1, MAGE-A3 and XAGE-1b mRNA expression, the results obtained depended on the HDACi used and on the CTA studied. Depending on the MPM cell line studied, molecules alone increased moderately PD-L1 expression. When combined, a higher stimulation of this immune check point inhibitor expression was observed. Decitabine-induced anti-viral response seemed to be inhibited in the presence of HDACi.

**Conclusions:**

This work shows that the combination of decitabine and HDACi could be of interest for MPM immunotherapy. However, this combination induced PD-L1 expression which suggests that an association with anti-PD-L1 therapy should be performed to induce an efficient anti-tumor immune response.

**Electronic supplementary material:**

The online version of this article (10.1186/s13148-018-0517-9) contains supplementary material, which is available to authorized users.

## Background

Malignant pleural mesothelioma (MPM) is a deadly disease that develops in the pleura. It was confirmed by Wagner’s studies that the vast majority of mesothelioma cases are actually attributed to asbestos exposure [1]. MPM was a very rare tumor before the industrial era; however, its incidence is increasing worldwide and is expected to peak in the year 2020. This cancer has an unusual molecular pathology with the loss of tumor suppressor genes being the predominant pattern of lesions [2]. Due to its resistance to all conventional therapies, the need for developing novel therapeutic regimens is urgent in order to cure this rare and treatment-resistant cancer.

In many cancers including mesothelioma, a hypermethylation of some gene promoters, including tumor suppressor genes (TSG) promoters, and overall DNA hypomethylation have been observed [3, 4]. Likewise, a decrease of acetylation of histones H3 and H4 was described [5, 6]. Therefore, epigenetics based therapies could be an interesting opportunity to improve MPM treatment. It was shown in different cancers that the use of hypomethylating agents in combination or not with histone deacetylase inhibitors (HDACi) could be immunogenic [7–11]. It has been shown, in the laboratory and in another team, that MPM cell death induced by the combination of a hypomethylating agent/HDAC inhibitor could be immunogenic through the induction of tumor antigen expression especially cancer testis antigens (CTA) [12, 13]. Even if epigenetic drugs are efficient especially as a treatment of hematopoietic cancers such as leukemia, they display poor clinical benefits on solid tumors. Moreover, HDACi are unspecific and toxic to healthy cells. In addition, they have side effects such as hematologic toxicity, decreasing the number of platelets and leukocytes [14]. It is, therefore, necessary to develop new epigenetic drugs that are more specific, less toxic to healthy cells and specifically for immune cells, and that can act in low doses.

In order to identify more potent and more convenient HDACi for clinic, we tested four new HDACi we recently synthetized and characterized [15, 16]. First of all, we evaluated the effect of these HDACi in comparison with valproic acid (VPA) and suberoylanilide hydroxamic acid (SAHA), two well-known HDACi, on total lymphocytes and on CD8+ T cell clones viability, and on some important populations of immune cells (natural killer (NK) and regulatory T (Treg) cells). Secondly, we tested the effect of the combination decitabine/new HDACi, in comparison with decitabine/VPA or SAHA combinations, on cancer testis antigens (CTA) expression, NY-ESO-1, MAGE-A1, MAGE-A3 and XAGE-1b, at the mRNA level in a malignant pleural mesothelioma cell lines. Indeed, CTA expression is associated with spontaneous antitumor immune responses and their discovery has led to the development of immunotherapy strategies and to antigen-specific cancer vaccines. Following treatment with decitabine/HDACi combinations, recognition of MPM cells by a NYESO-1-specific CD8+ T-cell clone was assessed. PD-L1 mRNA expression, a molecule that is implicated in the major mechanism of immuno-suppression within the tumor microenvironment [17], was also measured. Recently, it was demonstrated that hypomethylating agent can induce interferon pathway through activation of endogenous retroviral elements in colorectal, breast, and ovarian cancer [10, 11]. Therefore, the effect of the combinations was studied also on the mRNA expression of MDA5 (melanoma-differentiation-associated gene 5) and RIG-1 (retinoic-acid-inducible protein I), two essential immunoreceptors implicated in interferon signaling pathway and in RNA-sensing pathway.

## Methods

### Cell culture

The tumor cell lines used in this work were Meso96, Meso34, and Meso45, three human pleural mesothelioma cell lines, established from MPM patients’ effusions obtained by thoracocentesis (the patient was informed and gave signed consent). The cell lines were characterized and cultured in our laboratory as described [18]. Cells were cultured in Roswell Park Memorial Institute (RPMI) 1640 medium supplemented with 10% heat-inactivated fetal calf serum, 2 mM L-glutamine, 100 IU/ml penicillin, and 200 μg/ml streptomycin.

Lymphocytes were obtained from the clinical transfer platform (DTC, CIC-biothérapies Nantes) of Nantes Hospital (Nantes, France). CD8 T-cell clones used for this study, HLA-A*0201/NY-ESO-1(157–165)-specific CD8 T-cell clone was described previously [19].

### Drugs

Decitabine (5-azaCdR) and VPA were purchased from Sigma-Aldrich (Saint-Quentin Fallavier, France). SAHA was from Interchim (Montluçon, France). HDACi ODB, NODB, ODH, and NODH were synthesized by Dr. Philippe Bertrand as described previously [15]. Structure of the HDACi is provided as Additional file 1: Table S1.

### Cell treatment

For dose-response experiments, lymphocytes were seeded at 4 × 10^5^ cells/well in RPMI 1640 8% HS in flat-bottomed 96-well plates, and CD8 T-cell clones were seeded at 50 × 10^3^ cells/well in 180 μl RPMI 1640 8% HS containing 150 IU IL-2. The treatments were carried out 24 h after seeding by adding decitabine at 500 nM for 72 h; then, HDACi treatments were performed for additional 48 h.

For flow cytometry experiments, lymphocytes were seeded at 4 × 10^5^cells/well in a flat-bottomed 96-well plate in 180 μl RPMI 1640 8% HS. Cells were treated with different HDACi for 48 h.

For tumor cell culture, 10^5^ cells were seeded per well of 6-well plate in 2 ml of culture medium and then incubated at 37 °C for 24 h. Cells were then treated with a combination of 0.5 μM decitabine for 72 h followed by HDACi, VPA 5 mM, SAHA 2.5 μM, ODB 7.5 μM, NODB 2.5 μM, ODH 2.5 μM, and NODH 25 nM for 48 h.

### Cell viability test

After treatments, cell viability was measured using CellTiter-Glo kit (Promega) according to the manufacturer’s protocol.

### Flow cytometry analysis

The day of analysis, cells were stained in a conical-bottomed 96-well plate at 10^5^ cells/well. The plate was centrifuged at 800×*g* for 1 min, and the pellets were washed using 200 μl/well of PBS containing BSA 0.1% (wash buffer). Antibodies (see Additional file 2: Table S2) were used at a dilution of 1/30 in the wash buffer, and then, the plate was incubated at 4 °C for 30 min. Later, two washings were performed using the wash buffer prior to flow cytometry analysis. All flow cytometry data were acquired with FACScalibur (BD biosciences) using the CellQuest software (BD Biosciences) and analyzed by FlowJo software.

### Real-time RT-PCR

Expression levels of the gene of interest were analyzed using real-time PCR. Reverse transcription was performed with the M-MLV Reverse Transcriptase (Invitrogen) using aliquots of total RNA extracted from MPM cells NucleoSpin® RNA kits. All real-time PCR reactions were performed using the Mx3005P QPCR Systems (Stratagen Products, Agilent Technologies), and the amplifications were done using the SYBR Green PCR Master Mix SAB bioscience (Qiagen) mixed with Oligonucleotides QuantiTect Primer (Qiagen). The thermal cycling conditions were composed of 1 cycle at 95 °C for 10 min, 40 cycles at 95 °C for 30 s and 60 °C for 1 min, and 1 cycle at 95 °C for 1 min, 60 °C for 30 s, and 95 °C for 30 s. The experiments were carried out in duplicate for each data point. All the qPCR data were analyzed by MxPro software.

### Measurement of NY-ESO-1-specific CD8+ T-cells activation

MPM cells were treated or not with decitabine 72 h/HDACi 48 h prior to be seeded at 10^5^ cells/well and then co-cultured with NY-ESO-1-specific CD8+ T-cells [19] at 5 × 10^4^ cells/well in complete RPMI 1640 medium containing 10 mg mL^−1^ of brefeldin A (Sigma-Aldrich) for 6 h at 37 °C, then washed. Cells were stained with APC-conjugated mouse anti-human CD8 at 1/30 for 30 min at 4 °C in wash buffer, and PE-conjugated mouse anti-human IFN-γ monoclonal antibodies at 1/50 for 30 min at room temperature in permeabilization buffer. CD8 and IFN-γ expression were analyzed using flow cytometry.

### Statistical analysis

Data presented are means ± S.E.M. The unpaired *t* test and one-way ANOVA test followed by Holm-Sidak’s multiple comparisons test were used to measure the statistical differences. Statistical analyses were performed using GraphPad Prism 6 (GraphPad Software Inc., San Diego, CA, USA). A *P* value of 0.05 or less was considered as significant.

## Results

### Effect of histone deacetylase inhibitors (HDACi) on lymphocyte viability

The first step in this study was to test the toxicity of the novel compounds, in comparison with the two HDACi already known and used clinically (VPA and SAHA), on lymphocytes and on activated CD8 T lymphocytes clones. For this, we performed a cell viability assay after 48 h of treatment with increasing doses of HDACi on cells pretreated or not with decitabine. The IC_50_ were determined and summarized in Table 1, and the area under the curve (AUC) are provided as Additional file 3: Table S3.

**Table 1:** HDACi IC_50_ on immune cells

	Lymphocytes		T-CD8 clones	
	− Decitabine	+ Decitabine 500 nM	− Decitabine	+ Decitabine 500 nM
VPA	6.75 ± 0.06 mM	3.05 ± 0.11 mM	3.80 ± 0.06 mM	0.02 ± 0.10 mM
SAHA	13.66 ± 0.05 μM	9.9 ± 0.08 μM	1.29 ± 0.05 μM	0.08 ± 0.06 μM
ODB	ND	ND	28.81 ± 0.05 μM	0.17 ± 0.12 μM
NODB	46.01 ± 0.06 μM	26.22 ± 0.05 μM	17.99 ± 0.05 μM	0.37 ± 0.12 μM
ODH	9.62 ± 0.05 μM	5.28 ± 0.09 μM	1.86 ± 0.05 μM	0.07 ± 0.07 μM
NODH	0.43 ± 0.06 μM	0.19 ± 0.11 μM	0.03 ± 0.05 μM	0.002 ± 0.06 μM

IC_50_ values were determined using GraphPad prism, Prism 6 for Windows, by curve fitting using a sigmoidal dose response model. Results are the means ± S.E.M of three independent experiments

**Fig. 1:**
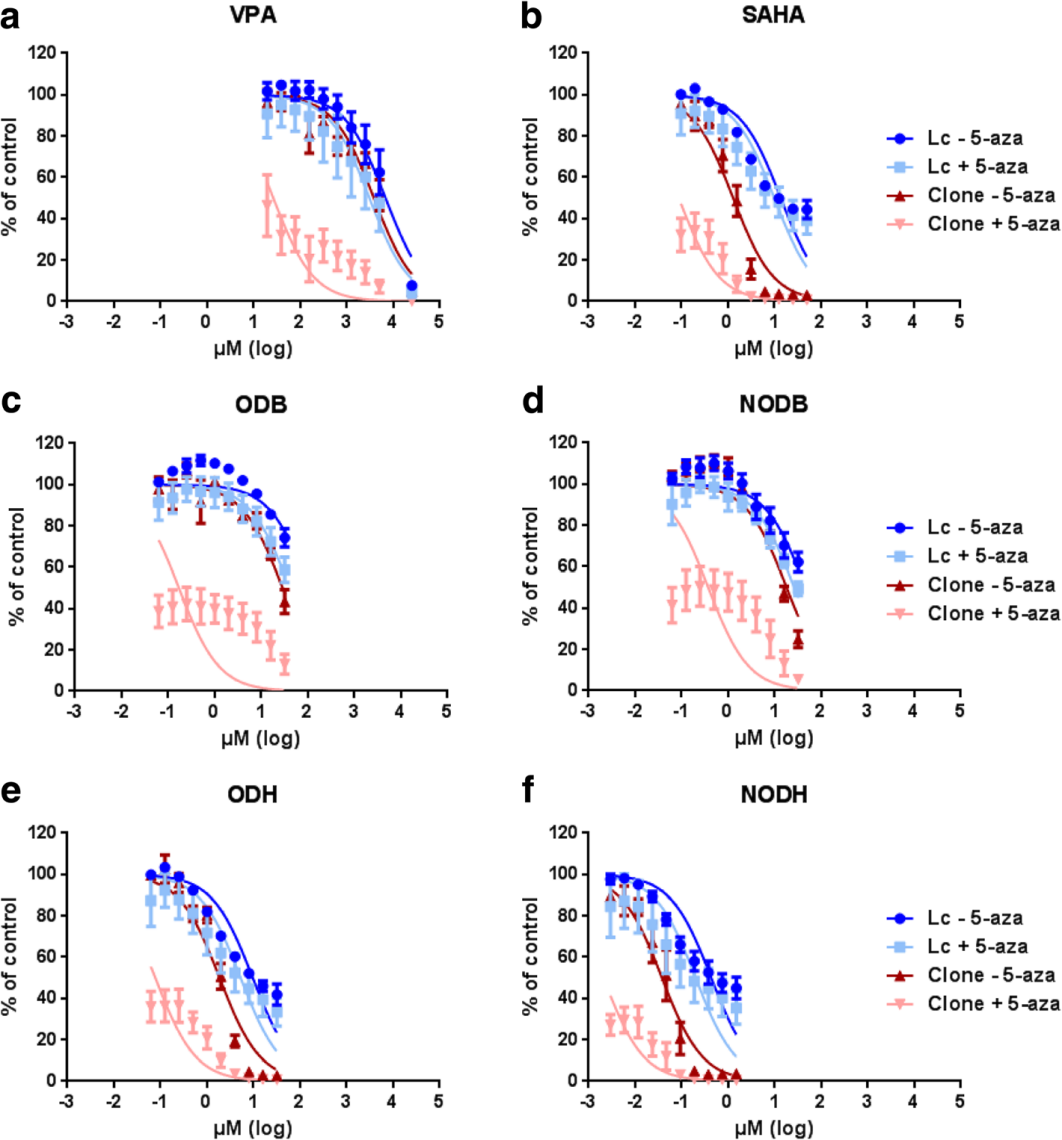
Effect of the different HDACi, in combination or not with decitabine, on lymphocytes and T-cell clones viability. Lymphocytes (Lc) and T-cell clones (Clone) were treated with increasing concentrations of VPA (**a**), SAHA (**b**), ODB (**c**), NODB (**d**), ODH (**e**), and NODH (**f**) for 72 h, in combination or not with decitabine (5-aza) 500 nM (72 h pretreatment). Viability was measured using Cell Titer-Glo kit. Results are expressed as the means ± S.E.M of three independent experiments

In Fig. 1, we can observe that the chemotherapeutic agents were toxic for lymphocytes and CD8+ T-lymphocyte clones at concentrations depending on compounds. For all tested molecules, IC_50_ were lower on CD8+ T-lymphocyte clones compared to total lymphocytes and are coherent with those we previously obtained on cancer cells [16]. Combination with decitabine did not increase drastically the toxicity of HDACi towards total lymphocytes (Fig. 1, Table 1, and Additional file 3: Table S3). However, on CD8 T-cell clones, IC_50_ of HDACi were strongly decreased in the presence of decitabine (Fig. 1, Table 1, and Additional file 3: Table S3). With lymphocytes, we note that some cells persist despite being treated with the highest concentration of compounds. Indeed, in the presence or not of decitabine, approximately 40% of the cells survived with 5 mM of VPA and with the highest doses of SAHA, ODH, and NODH, and about 50–60% of the cells survived with the highest dose of ODB and NODB.

### Histone deacetylase inhibitors effect on immune cells subpopulations

The aim of our strategy is to induce an anti-tumor immune response to cure malignant pleural mesothelioma by using combinations of epigenetic drugs including HDACi. Therefore, it was necessary to study the effect of these HDACi on sub-populations of lymphocytes implicated in this immune response. Doses of compounds were chosen according to blood concentrations of VPA and SAHA measured during clinical trials [20–23] and to induce approximately 50% of decrease of cell viability for ODB, NODB, ODH, and NODH on total lymphocytes (Fig. 1). Toxicity of the compounds was tested on natural killer cells (NK) (CD56+, CD16+), on regulatory T-cells (Treg) (CD4+, CD25+, CD127 low and Foxp3+), on CD4 and CD8 naïve T-cells (CD4+, CCR7+ and CD45RA+, and CD8+, CCR7+ and CD45RA+, respectively), and on CD4 and CD8 memory T cells (CD4+ and CD45RO+, and CD8+ and CD45RO+, respectively).

**Fig. 2:**
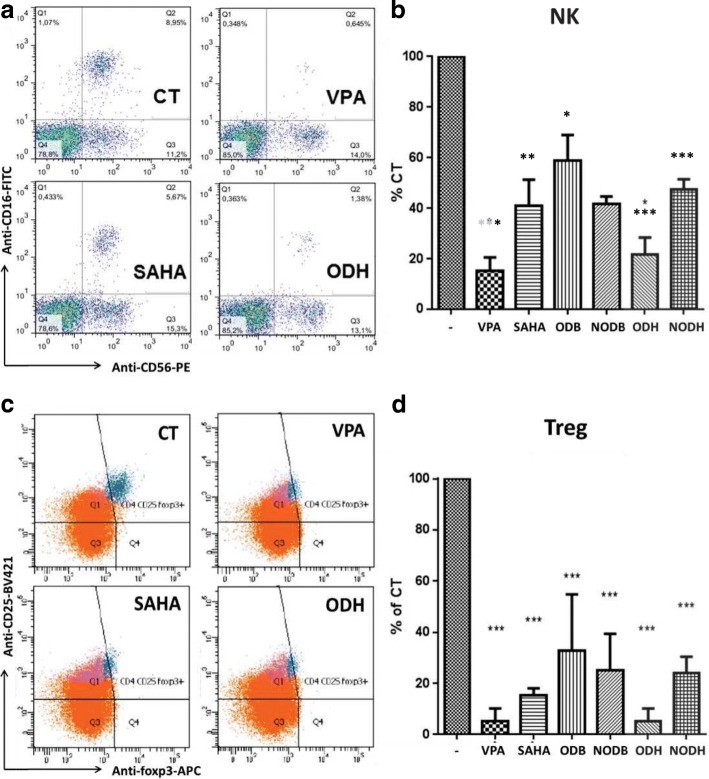
Effect of HDAC inhibitors on NK and Treg cells. Lymphocytes obtained by elutriation were treated with HDACi at the following concentrations for 48 h: VPA 5 mM, SAHA 1 μM, ODB 32 μM, NODB 8 μM, ODH 4 μM, and NODH 50 nM. **a** and **c** are examples of results obtained on natural killer cells (NK) and regulatory T cells (Treg) using flow cytometry. **b** and **d** are graphic representations of HDACi effect on NK and Treg proportions (respectively). Results are expressed as the means ± S.E.M of three independent experiments. *p < 0.05, **p < 0.01 and ***p < 0.001

The proportion of NK cells was decreased by almost all HDACi and more particularly by ODH and VPA (more than 50% in comparison to the untreated lymphocytes) (Fig. 2a, b). Then, we analyzed the effect of the molecules on Treg cells, a subset of T lymphocytes implicated in the inhibition of the immune response. Figure 2c, d shows that VPA and ODH decreased Treg by approximately 95%, while the other compounds reduced the proportion of this sub-population by 85 to 70% approximately. The populations of naïve and memory T-cells were not significantly affected by the different HDACi (Additional file 4: Figure S1).

### Effect of HDACi in combination with decitabine on cancer testis antigens expression in mesothelioma cells

**Fig. 3:**
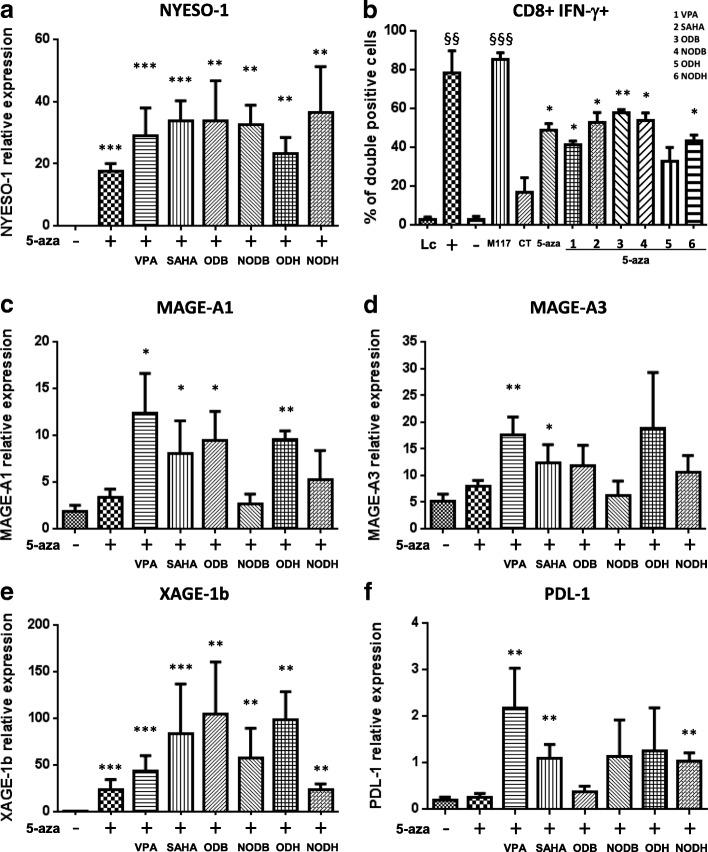
HDACi increase decitabine-induced CTA expression in Meso96 cells and allow tumor cell recognition by CD8+ NY-ESO-1 T-cell clone. Meso96 cells were treated with: VPA 5 mM, SAHA 2.5 μM, ODB 7.5 μM, NODB 2.5 μM, ODH 2.5 μM, and NODH 25 nM (48 h) in combination or not with decitabine (5-aza) 500 nM (72 h pretreatment). NY-ESO-1 (**a**), MAGE-A1 (**c**), MAGE-A3 (**d**), XAGE-1b (**e**), and PD-L1 (**f**) mRNA were measured using real time PCR. *p < 0.05, **p < 0.01 and ***p < 0.001. **b** Interferon (IFN-γ) production by NY-ESO-1-specific CD8+ T-cell clone in response to Meso96 treated or not with the combination decitabine +/− HDACi. IFN-γ production was measured by intracytoplasmic staining of IFN-γ and surface staining of CD8, followed by flow cytometry analysis. Lc: lymphocytes alone, +: NY-ESO-1 (157–165) peptide (10 μM), −: MUC1 (950–958) peptide (10 μM), M117: melanoma cell line that expresses NY-ESO-1, CT: untreated Meso96 cells. Results are expressed as the means ± S.E.M of three independent experiments. § vs LC, ^§§^*p* < 0.001, ^§§§^*p* < 0.001; * vs CT, *p < 0.05 and **p < 0.01

Cancer testis antigens (CTA) are a category of tumor antigens that are highly restricted to tumors. Their expression is associated with spontaneous antitumor immune response, and their discovery has led to the development of immunotherapy strategies and to antigen-specific cancer vaccines [24]. It was demonstrated that CTA expression can be induced using combination of decitabine/HDAC inhibitor. We and others previously demonstrated that CTA can be induced in mesothelioma cells using this strategy [12, 13]. Here, we aimed at evaluating the immunogenicity of the new HDACi in combination with decitabine. For this work, we used the mesothelioma cell lines Meso96, Meso34, and Meso45, established and characterized in the laboratory [18]. These treatments were poorly toxic on Meso96 and Meso34 (Additional file 5: Figure S2). However, on Meso45, molecules used alone induced from 10 to 60% of decrease of cell viability. With HDACi in combination with decitabine, the decrease of cell viability was from 60 to 90% (Additional file 5: Figure S2). Figure 3a, b shows the effect of the new compounds in combination with decitabine on the mRNA expression of the CTA, NY-ESO-1 (Fig. 3a), and the recognition of the treated cells by a CD8 T-cell clone specific of this antigen (Fig. 3b). HDACi alone have no effect on CTA expression (Additional file 6: Figure S3). All HDACi increased significantly the mRNA expression of NY-ESO-1 induced by decitabine. This effect was associated with recognition of the treated mesothelioma cells by the HLA-A*0201/NY-ESO-1(157–165)-specific CD8 T-cell clone, characterized by the production of IFN-γ after co-culture experiments. This result was not associated with an increase of HLA molecules at mesothelioma cell surface which demonstrated that recognition by T-cell clone was driven by the increase of NY-ESO-1 expression (Additional file 7: Figure S4). We extended this study to the mRNA expression of other CTA, MAGE-A1 (Fig. 3c), MAGE-A3 (Fig. 3d) and XAGE-1b (Fig. 3e). Globally, all HDACi increased CTA induced by decitabine. However, some differences can be observed depending on the CTA and on the molecule evaluated. Similar results were obtained using two additional MPM cell lines, Meso34 and Meso45 (Additional file 8: Figure S5 and Additional file 9: Figure S6). We also assessed the effect of these HDAC inhibitors on the mRNA expression of PD-L1 (B7-H1), an immunomodulatory molecule that inhibits T cell-mediated immune response [25]. Whereas decitabine alone did not affect the mRNA expression of PD-L1, when combined with VPA, SAHA, or NODH, a significant increase of PD-L1 mRNA was observed (Fig. 3f). In the two additional MPM cell lines, decitabine, some HDACi alone, VPA, SAHA and ODH in particular, and all the combinations increased PD-L1 expression (Additional file 8: Figure S5 and Additional file 9: Figure S6).

### Effect of decitabine alone and/or in combination with HDACi on RIG-1 and MDA5 mRNA expression in mesothelioma cells

**Fig. 4:**
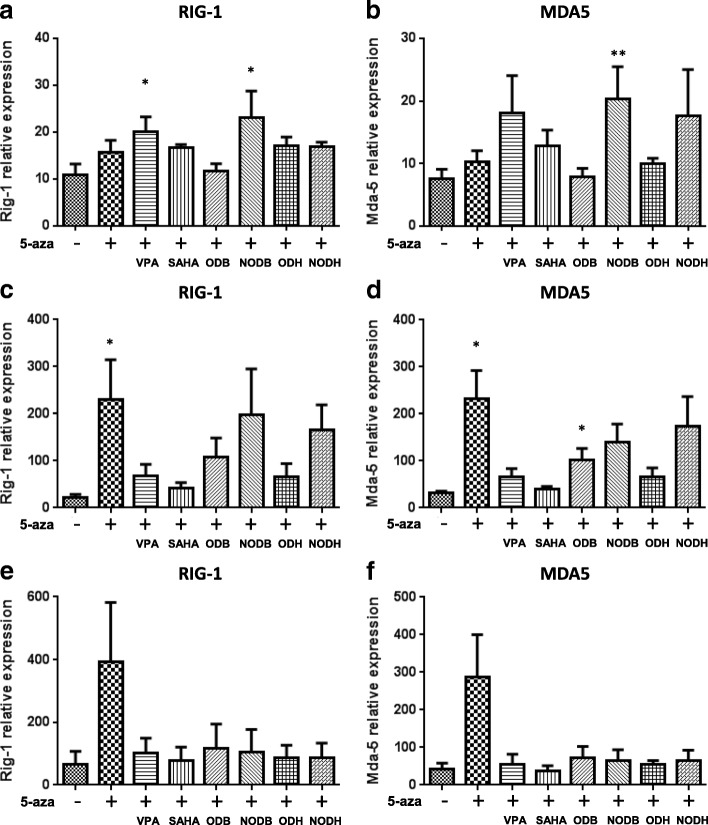
HDACi modulate decitabine-induced RIG-1 and MDA5 expression in MPM cells. Meso96 (**a**, **b**), Meso34 (**c**, **d**), and Meso45 (**e**, **f**) cells were treated with: VPA 5 mM, SAHA 2.5 μM, ODB 7.5 μM, NODB 2.5 μM, ODH 2.5 μM, and NODH 25 nM (48 h) in combination or not with decitabine (5-aza) 500 nM (72 h pretreatment). RIG-1 (**a**, **c**, and **e**) and MDA5 (**b**, **d**, and **f**) mRNA expression were measured using real time PCR. *p < 0.05 and **p < 0.01

Several works demonstrated that decitabine can induce interferon-responsive genes such as melanoma differentiation-associated antigen-5 (MDA5) and retinoic acid-inducible gene I (RIG-1) in different cancer cell lines [7, 10, 11, 26]. The induction of this pathway can lead to cell cycle arrest. In MPM cells, the treatment with decitabine alone increased significantly the expression of both RIG-1 and MDA5 mRNA expression in Meso34 (Fig. 4c, d) and Meso45 (Fig. 4e, f) but not in Meso96 (Fig. 4a, b). HDACi alone induced no modifications of RIG-1 and MDA5 expression in all tested cell lines (Additional file 10: Figure S7). Decitabine in combination with HDACi had negligible effect on RIG-1 and MDA5 expression in Meso96 (Fig. 4a, b); however, RIG-1 and MDA5 expression induced by decitabine was reduced in Meso34 (Fig. 4c, d) and Meso45 (Fig. 4e, f).

## Discussion

Malignant pleural mesothelioma (MPM) is a locally invasive and rapidly fatal malignancy often diagnosed 30–40 years after exposure to asbestos. To date, there is no effective therapeutic strategy against this disease; therefore, the development of new treatment is required. The use of epigenetic drugs represents a promising approach to treat people suffering from MPM. The hypomethylating drug decitabine (5-azaCdR) and HDAC inhibitors have many positive effects either by promoting growth arrest, apoptosis or differentiation of tumor cells [27, 28]. In a previous work on a mice model of mesothelioma, we have shown that the use of these drugs alone did not have a strong anti-tumoral effect, while the use of the combination of decitabine/HDAC inhibitors, VPA or SAHA, was efficient [12]. The use of these FDA-approved drugs had an impact on the expression of CTA in mesothelioma cells and induced an anti-tumor immune response in vivo. However, these compounds have many toxic properties, notably hematologic toxicity, which requires the development of new HDAC inhibitors that are less toxic to healthy cells, act at low doses and preserve immune cells.

In this study, we aimed at characterizing the toxicity of new HDACi, in comparison with VPA and SAHA, on immune cells. Then, we studied their immunogenicity, in combination with decitabine, on MPM cells by measuring the mRNA expression of CTA, PD-L1, MDA5, and RIG-1. Our results showed that toxicity on immune cells and immunogenicity on MPM cells of the tested molecules are different. This study demonstrates that a careful evaluation of HDACi is required to define the best combination strategy for immunotherapy application.

All the tested molecules were toxic for immune cells. However, whereas high doses of HDACi led to the death of 100% of CD8 T-cells clones on total lymphocytes, maximal toxicity depended on the molecule used. When compared to blood concentrations reach in clinic for SAHA, approximately 1 μM [22, 23], these results are coherent with the hematological toxicity observed in patients. Concerning VPA, blood concentrations observed during treatments are around 1 mM [20, 21]. At this concentration, VPA presents weak hematological toxicity according to our observation. Extrapolated to our new compounds, NODH seems to be the more promising. Indeed, this HDACi is 100 times more active to induce histone H3 acetylation in cells than SAHA [29]. Whereas SAHA is toxic for both lymphocytes and CD8 T-cells at clinical doses measured in blood from patients (approximately 1 to 4 μM) [30, 31], NODH at 10 to 40 nM, 100 times lower concentrations than SAHA, was poorly toxic.

Additional analyses demonstrated that Treg and NK cell populations were particularly affected by HDACi. All the tested compounds reduced significantly the proportion of Treg. However, VPA and ODH compounds were the most active drugs on these cells. Epigenetic mechanisms could be responsible for these effects. There is no evidence of a regulation of Treg and NK cell markers through modulation of histone acetylation. FoxP3, a strong marker of Treg, is well-known to be regulated by methylation [32], and recent study suggests that CD16 expression could also be regulated by methylation [33]. Decitabine alone reduced moderately Treg and NK proportion (Additional file 11: Figure S8). However, here, it is difficult to determine whether the effect was more a toxicity or an epigenetic regulation of cell markers. A mechanistic study is necessary to identify mechanisms involved.

In clinic, the effect of HDACi on Treg cell population is not clear. Some studies mentioned a decrease of Treg cell population following HDACi treatment [34, 35] whereas others reported an increase of Treg number and function [36]. In vitro and preclinical models suggest that reduction of Treg cells by HDACi depends on the specificity and on the dose of the molecules used [37]. According to the implication of Treg cells in the inhibition of anti-tumor immune response, this effect of HDACi could be beneficial for immunotherapy strategy. NK cells are implicated in innate immunity. HDACi were already described as toxic for these particular cells [38–40], as observed in our study. This action of HDACi could be deleterious for induction of anti-tumor response in vivo regarding the implication of NK cells in tumor immune-surveillance [41, 42] and metastasis control [43]. However, on the contrary of Treg cells, the impact of HDACi on NK cell proportion is more moderate. Indeed, Treg cells were reduced from 65 to 95% and NK cells were reduced from 40 to 85%.

Several studies have demonstrated the immunogenicity of the combination decitabine/HDACi on cancer cells, notably on mesothelioma cells [12, 13]. In this study, we showed that all HDACi tested increased decitabine-induced CTA expression which was associated with an increase activation of a NY-ESO-1-specific CD8 T cell in vitro. This result was not associated with an increase of HLA molecules at mesothelioma cell surface (Additional file 7: Figure S4) which demonstrated that the recognition by T cell clone was driven by the increase of NY-ESO-1 expression. We previously demonstrated that HDAC1 is in part responsible for NY-ESO-1 repression [44]. Thus, an action of all tested HDACi on decitabine-induced NY-ESO-1 expression was expected according to their inhibitory activity on HDAC1 (Additional file 12: Figure S9). For the other CTA tested, there is no available information on the HDAC implicated in their regulation. Additionally, we observed that decitabine increased RIG-1 and MDA5 expression in Meso34 and Meso45 whereas in Meso96 the modifications observed were negligible. This observation is novel in the field of MPM. RIG-1 and MDA5 are two receptors implicated in interferon signaling pathway and viral infection response. The increase of RIG-1 and MDA5 expression by decitabine was described as the consequence of the induction of endogenous virus replication and then of the activation of the interferon signaling pathway [10, 11]. In Meso34 and Meso45, combination with HDACi reduced induction of RIG-1 and MDA5 expression by decitabine. Anti-replicative effect of HDACi on viruses was already described [45] which could explain the results obtained. Thus, immunogenicity of decitabine could be reduced. However, induction of endogenous viruses by decitabine could also be responsible for an increase of the mutation rate in MPM cells and then to an improve adaptability to their environment. Regarding our previous results obtained in vivo with the combination decitabine/HDACi [12], it seems that inhibition of interferon signaling pathway induced by decitabine is not determinant for the induction of an anti-tumor response. Moreover, recent data from our laboratory has demonstrated that approximately 70% of MPM have a non-functional interferon pathway (Achard et al., Oncotarget, 2015).

Recently, immune check point inhibitors were identified as responsible for inhibition of anti-tumor immune response and then appear as promising therapeutic targets [17]. The PD-1/PD-L1 axis is of particular interest regarding the impressive results observed in clinic with anti-PD-1 blocking antibody. Therefore, the impact of treatments on PD-L1 expression in cancer cells needs to be monitored to avoid a fail of the strategy evaluated. Indeed, several studies demonstrated that the expression of PD-L1 can be increased in cancer cells following HDACi or hypomethylating agent treatment [46]. In MPM cells, we observed that PD-L1 was induced by the combination decitabine/HADCi. This observation suggests that epigenetic regulation of CTA expression and PD-L1 could be associated.

In view of our results, it seems that there is no really more appropriate molecule when regarding immunogenicity. Our study suggests that the combination decitabine/HDACi should be associated with anti-PD-L1 strategy given that all combinations induced PD-L1 gene expression. However, NODH could be interesting regarding its pharmacological properties. Indeed, this HDACi is active at nanomolar concentrations and at concentrations which induce gene expression, NODH is moderately toxic for lymphocytes when combined with decitabine (25% of cell death). Unfortunately, toxicity on T-cell clone should be considered in the protocol of treatment to avoid hampering an ongoing anti-tumor immune response.

## Conclusions

From our work, it seems that more careful studies are needed on the intrinsic properties of HDACi on both immune and cancer cells. Indeed, data obtained will be useful to define the most appropriate combination of molecules for immunotherapy strategies. Likewise, a better comprehension of molecular players participating in the regulation of genes implicated in immunogenicity is required to design optimized HDACi. To date, anti-PD-1/PD-L1 strategy needs to be considered in combination with decitabine and HDACi for MPM treatment in order to overcome a possible induction of PD-L1 expression.

## Additional files

Additional file 1: **Table S1.** Structure and class of the compounds used in this study. (DOCX 37 kb)

Additional file 2: **Table S2.** Detailed information about the antibodies used in the experiments. (DOCX 16 kb)

Additional file 3: **Table S3.** HDACi AUC on immune cells. (DOCX 14 kb)

Additional file 4: **Figure S1.** Effect of HDAC inhibitors on naïve and memory T-cells. Lymphocytes obtained by elutriation were treated with HDACi at the following concentrations for 48 h: VPA 5 mM, SAHA 1 μM, ODB 32 μM, NODB 8 μM, ODH 4 μM, and NODH 50 nM. Graphics represent the effect of the compounds on CD4 (a) and CD8 (b) naïve T-cells and on CD4 (c) and CD8 (d) memory T-cells. Results are expressed as the means ± S.E.M of three independent experiments. (PDF 108 kb)

Additional file 5: **Figure S2.** Effect of decitabine and HDACi, in combination or not, on MPM cell growth. MPM cells were treated with: VPA 5 mM, SAHA 2.5 μM, ODB 7.5 μM, NODB 2.5 μM, ODH 2.5 μM, and NODH 25 nM (48 h) in combination or not with decitabine (5-aza) 500 nM (72 h pretreatment). Viability was measured using Cell Titer Glo kit (Promega). *p < 0.05, **p < 0.01 and ***p < 0.001. (PDF 179 kb)

Additional file 6: **Figure S3.** HDACi increase decitabine-induced CTA expression in Meso96 cells. Meso96 cells were treated with: VPA 5 mM, SAHA 2.5 μM, ODB 7.5 μM, NODB 2.5 μM, ODH 2.5 μM, and NODH 25 nM (48 h) in combination or not with decitabine (5-aza) 500 nM (72 h pretreatment). NY-ESO-1, MAGE-A1, MAGE-A3, XAGE-1b and PD-L1 mRNA were measured using real time PCR. (PDF 199 kb)

Additional file 7: **Figure S4.** Expression of HLA ABC in Meso96 treated with decitabine/HDACi combinations. Meso96 were treated with: VPA 5 mM, SAHA 2.5 μM, ODB 7.5 μM, NODB 2.5 μM, ODH 2.5 μM, and NODH 25 nM (48 h) in combination or not with decitabine (5-aza) 500 nM (72 h pretreatment). Then, HLA ABC expression was measured using flow cytometry. Results are expressed as the means ± S.E.M of three independent experiments. (PDF 91 kb)

Additional file 8: **Figure S5.** HDACi increase decitabine-induced CTA expression in Meso34 cells. Meso34 cells were treated with: VPA 5 mM, SAHA 2.5 μM, ODB 7.5 μM, NODB 2.5 μM, ODH 2.5 μM, and NODH 25 nM (48 h) in combination or not with decitabine (5-aza) 500 nM (72 h pretreatment). NY-ESO-1, MAGE-A1, MAGE-A3, XAGE-1b and PD-L1 mRNA were measured using real time PCR. (PDF 197 kb)

Additional file 9: **Figure S6.** HDACi increase decitabine-induced CTA expression in Meso45 cells. Meso45 cells were treated with: VPA 5 mM, SAHA 2.5 μM, ODB 7.5 μM, NODB 2.5 μM, ODH 2.5 μM, and NODH 25 nM (48 h) in combination or not with decitabine (5-aza) 500 nM (72 h pretreatment). NY-ESO-1, MAGE-A1, MAGE-A3, XAGE-1b and PD-L1 mRNA were measured using real time PCR. (PDF 197 kb)

Additional file 10: **Figure S7.** HDACi modulate decitabine-induced RIG-1 and MDA5 expression in MPM cells. Meso34 (top), Meso45 (middle) and Meso96 (down) cells were treated with: VPA 5 mM, SAHA 2.5 μM, ODB 7.5 μM, NODB 2.5 μM, ODH 2.5 μM, and NODH 25 nM (48 h) in combination or not with decitabine (5-aza) 500 nM (72 h pretreatment). RIG-1 (left) and MDA-5 (right) mRNA expression were measured using real time PCR. *p < 0.05, **p < 0.01 and ***p < 0.001. (PDF 209 kb)

Additional file 11: **Figure S8.** Effect of decitabine on Treg and NK cells. Lymphocytes obtained by elutriation were treated with decitabine (5-aza) 500 nM (72 h). Figure are examples of results obtained on A) natural killer cells (NK) and B) regulatory T cells (Treg) using flow cytometry. (PDF 281 kb)

Additional file 12: **Figure S9.** Determination of HDAC1 inhibition properties of ODB, NODB, ODH and NODH. Recombinant HDAC1 activity in the presence of increasing doses of ODB, NODB, ODH and NODH were measured using Fluor de Lys® Drug Discovery Assays (Enzo Life Sciences). (PDF 89 kb)

## Abbreviations

5-azaCdR: 5-Aza-2′-deoxycytidine
APC: Allophycocyanine
AUC: Area under the curve
BSA: Bovine serum albumin
CD4, CD8...: Cluster of differentiation 4, 8...
CT: Control
CTA: Cancer testis antigen
DNA: Deoxyribonucleic Acid
DTC Platform: ‘Développement et Transfert Clinique’ platform
FACS: Fluorescence Activated Cell Sorting
FITC: Fluorescence Iso Thio Cyanate
FoxP3: Forkhead box P3
HDACi: Histone deacetylase inhibitor
HLA: Human Leukocyte Antigens
HS: Human Serum
IC50: The half maximal inhibitory concentration
IFN-γ: Interferon-γ
IgG1: Immunoglobulin (antibody) G1
IL-2: Interleukin-2
MAGE-A1, MAGE-A3: Melanoma antigen-encoding genes family A
MDA5: Differentiation-associated antigen-5
M-MLV: Moloney Murine Leukemia Virus
MPM: Malignant pleural mesothelioma
mRNA: Messenger Ribonucleic Acid
MUC1: Mucin1
NK: Natural killer cells
NODB: 5-(6-Dimethylamino-2-methyl-3-oxo-2,3-dihydrobenzofuran-2-yl)-4methyl-penta-2,4-dienoic acid benzamide
NODH: 5-(6-Dimethylamino-2-methyl-3-oxo-2,3-dihydro benzofuran-2-yl)-4methyl-penta-2,4-dienoic acid hydroxamide
NY-ESO-1: New York Oesophageal Squamous Cell Carcinoma 1
ODB: 4-Methyl-5-(2-methyl-3-oxo-2,3-dihydro-benzofuran-2-yl)-penta-2,4-dienoic acid benzamide
ODH: 4-Methyl-5-(2-methyl-3-oxo-2,3-dihydro-benzofuran-2-yl)-penta-2,4-dienoic acid hydroxamide
PBS: Phosphate-Buffered Saline
PD-1: Programmed cell death 1
PD-L1: Programmed death-ligand 1
PE: Phycorerythrine
PerCP: Peridinin-chlorophyll-protein Complex Conjugate
RIG-1: Retinoic acid-inducible gene I
RMFI: relative median fluorescent intensity
RNA: Ribonucleic Acid
RPLPO: Ribosomal Protein Lateral Stalk Subunit P0
RPMI 1640 medium: Roswell Park Memorial Institute 1640 medium
RT-PCR: Real Time-Polymerase Chain Reaction
S.E.M: Standard Error of the Mean
SAHA: Suberoylanilide hydroxamic acid
T-reg: Regulatory T cells
TSG: Tumor suppressor genes
VPA: Valproate
XAGE-1b: X antigen family member 1b
