# Supplementary material for: Assessment of new HDAC inhibitors for immunotherapy of malignant pleural mesothelioma
Source: Clin Epigenetics. 2018 Jun 18;10:79. doi: 10.1186/s13148-018-0517-9 (PMC6006850; doi:10.1186/s13148-018-0517-9)
Supplement: Supplementary file 1 — Table S1. Structure and class of the compounds used in this study. (DOCX 37 kb) [file 13148_2018_517_MOESM1_ESM.docx]

**Table S1:** Structure and class of the compounds used in this study

| HDACi | Structure | Class |
| --- | --- | --- |
| VPA | 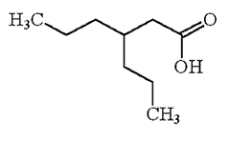 | Short chain fatty acid |
| SAHA | 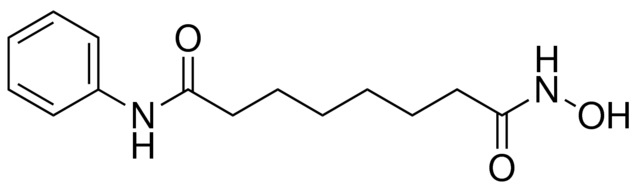 | Hydroxamic acid |
| ODB | **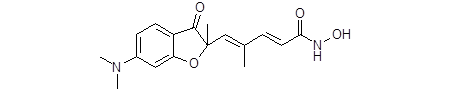** | Benzamide |
| NODB | 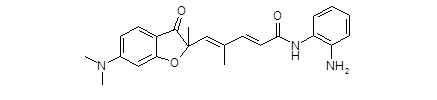 | Hydroxamic acid |
| ODH | 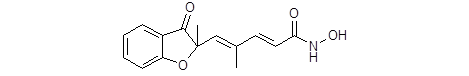 | Benzamide |
| NODH | 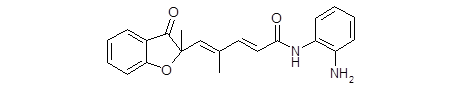 | Hydroxamic acid |
