# Supplementary material for: Assessment of new HDAC inhibitors for immunotherapy of malignant pleural mesothelioma
Source: Clin Epigenetics. 2018 Jun 18;10:79. doi: 10.1186/s13148-018-0517-9 (PMC6006850; doi:10.1186/s13148-018-0517-9)
Supplement: Supplementary file 2 — Table S2. Detailed information about the antibodies used in the experiments. (DOCX 16 kb) [file 13148_2018_517_MOESM2_ESM.docx]

**Table S2:** Detailed information about the antibodies used in the experiments

| Antibody | **Manufacturer** | **Fluorophore** | **Fluorescence** |
| --- | --- | --- | --- |
| CD4, CD16 | BD Biosciences | FITC | FL-1 |
| CCR7, CD274, HLA-A2, HLA-ABC, IFN-γ, & IgG-1 | BD Biosciences | PE | FL-2 |
| CD56 | BioLegend | PE/Cy5 | FL-3 |
| CD45RO | BioLegend | PerCP/Cy5.5 | FL-3 |
| CD45RA | BD Biosciences | FITC | FL-3 |
| CD4, CD8 | BioLegend | APC | FL-4 |

Antibodies used for the labeling of immune cells and in NY-ESO-1-specific CD8+ T-cells clone activation (FACS analysis)

| Antibody | **Manufacturer** | **Fluorophore** |
| --- | --- | --- |
| CD3 | BD Biosciences | APC-H7 |
| CD4 | BD Pharmingen | FITC |
| CD127 | BD Biosciences Pharmingen | PE |
| CD25 | BD Biosciences | BV421 |
| Foxp3 | BD Biosciences | AF647 |

Antibodies used for Treg Labeling (Canto analysis)
