# Supplementary material for: Assessment of new HDAC inhibitors for immunotherapy of malignant pleural mesothelioma
Source: Clin Epigenetics. 2018 Jun 18;10:79. doi: 10.1186/s13148-018-0517-9 (PMC6006850; doi:10.1186/s13148-018-0517-9)
Supplement: Supplementary file 3 — Table S3. HDACi AUC on immune cells. (DOCX 14 kb) [file 13148_2018_517_MOESM3_ESM.docx]

|  | **Lymphocytes** | | **T-CD8 clones** | |
| --- | --- | --- | --- | --- |
|  | **- 5-aza** | **+ 5-aza 500nM** | **- 5-aza** | **+ 5-aza 500nM** |
| **VPA** | 248.3 | 208.5 | 216.8 | 60.52 |
| **SAHA** | 200.5 | 183.9 | 112.2 | 35.07 |
| **ODB** | 275.9 | 239.5 | 238.6 | 95.24 |
| **NODB** | 258.0 | 231.2 | 243.1 | 101.7 |
| **ODH** | 203.6 | 178.3 | 147.1 | 47.10 |
| **NODH** | 197.5 | 169.5 | 111.0 | 32.34 |

**Table S3:** HDACi AUC on immune cells

AUC (Area Under the Curve) values were determined using GraphPad prism, Prism 6 for Windows. Results are the means ± S.E.M of three independent experiments.
