# Supplementary figures and images for: Assessment of new HDAC inhibitors for immunotherapy of malignant pleural mesothelioma
Source: Clin Epigenetics. 2018 Jun 18;10:79. doi: 10.1186/s13148-018-0517-9 (PMC6006850; doi:10.1186/s13148-018-0517-9)

Fig. S1

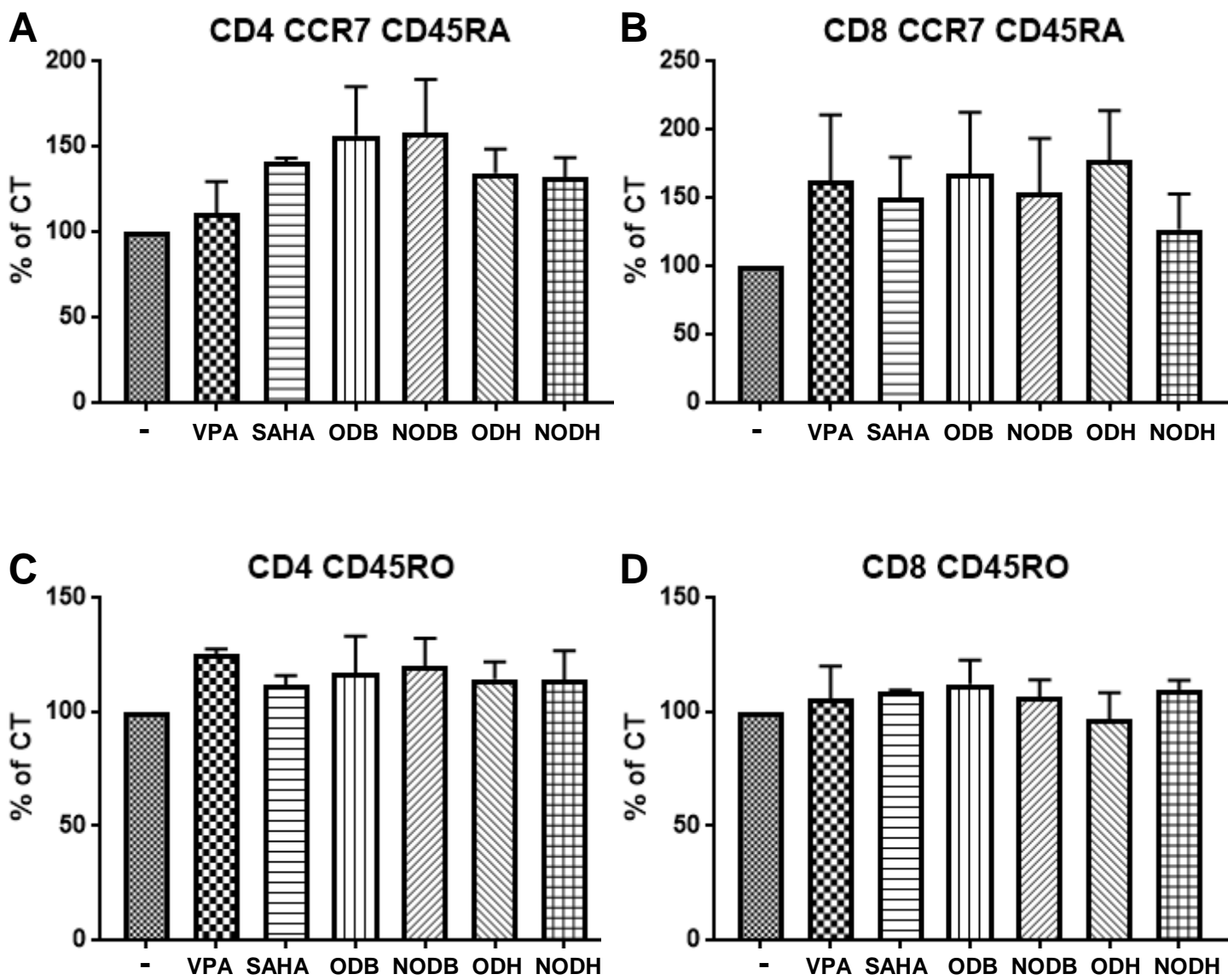

Supplement: Supplementary file 4 — Figure S1. Effect of HDAC inhibitors on naïve and memory T-cells. Lymphocytes obtained by elutriation were treated with HDACi at the following concentrations for 48 h: VPA 5 mM, SAHA 1 μM, ODB 32 μM, NODB 8 μM, ODH 4 μM, and NODH 50 nM. Graphics represent the effect of the compounds on CD4 (a) and CD8 (b) naïve T-cells and on CD4 (c) and CD8 (d) memory T-cells. Results are expressed as the means ± S.E.M of three independent experiments. (PDF 108 kb) [file 13148_2018_517_MOESM4_ESM.pdf]

Fig. S2

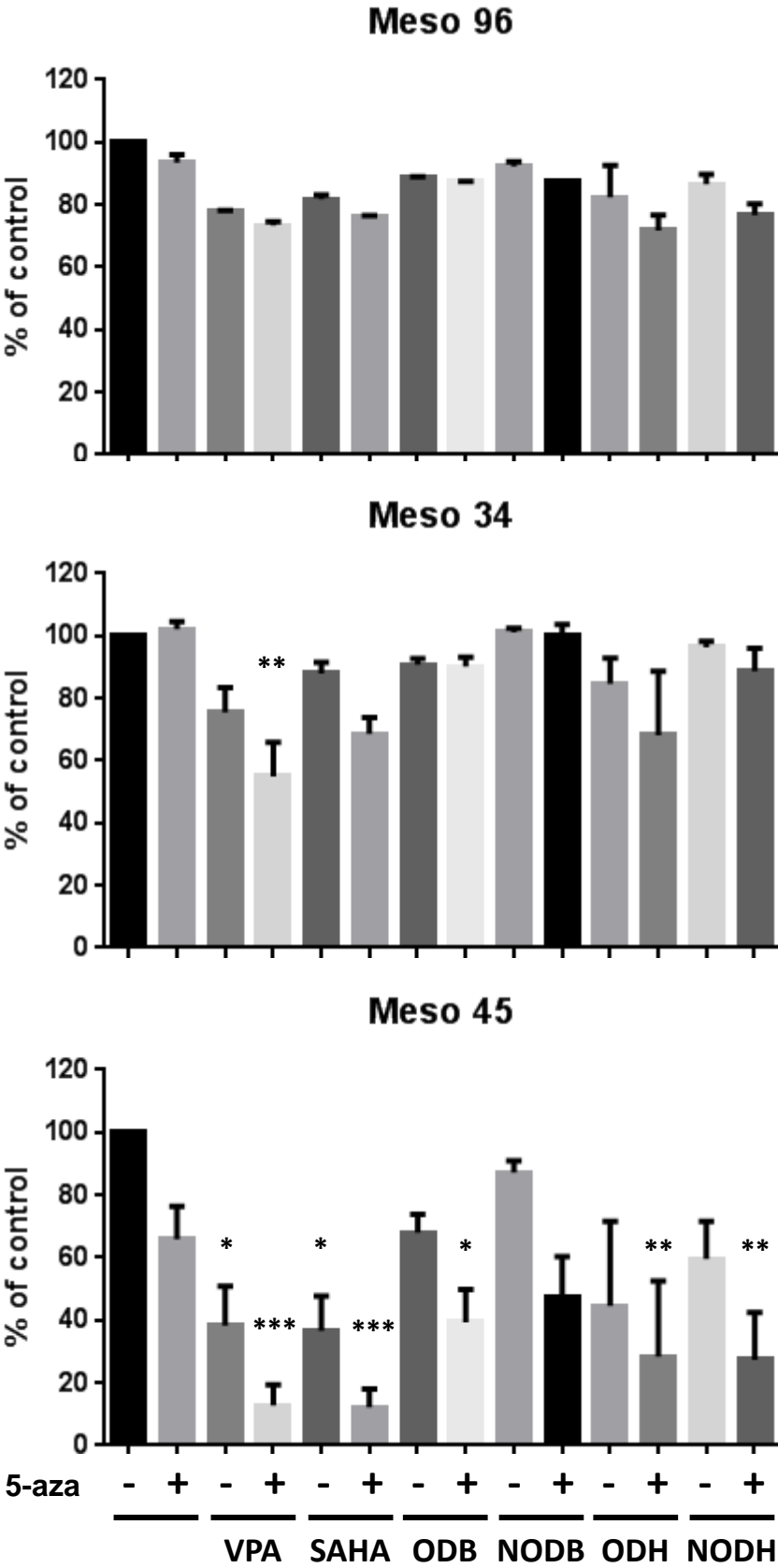

Supplement: Supplementary file 5 — Figure S2. Effect of decitabine and HDACi, in combination or not, on MPM cell growth. MPM cells were treated with: VPA 5 mM, SAHA 2.5 μM, ODB 7.5 μM, NODB 2.5 μM, ODH 2.5 μM, and NODH 25 nM (48 h) in combination or not with decitabine (5-aza) 500 nM (72 h pretreatment). Viability was measured using Cell Titer Glo kit (Promega). *p < 0.05, **p < 0.01 and ***p < 0.001. (PDF 179 kb) [file 13148_2018_517_MOESM5_ESM.pdf]

Fig. S3

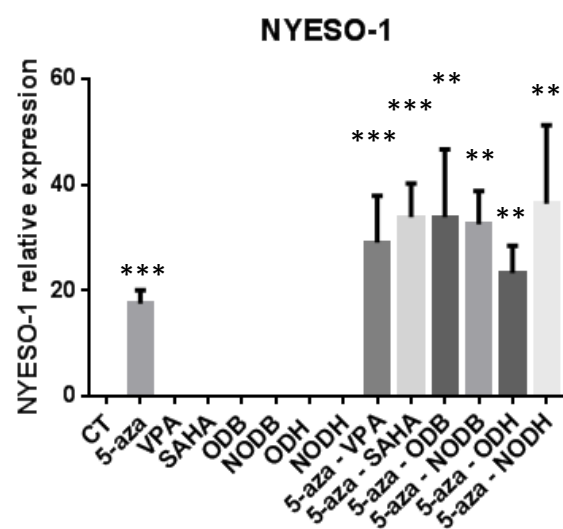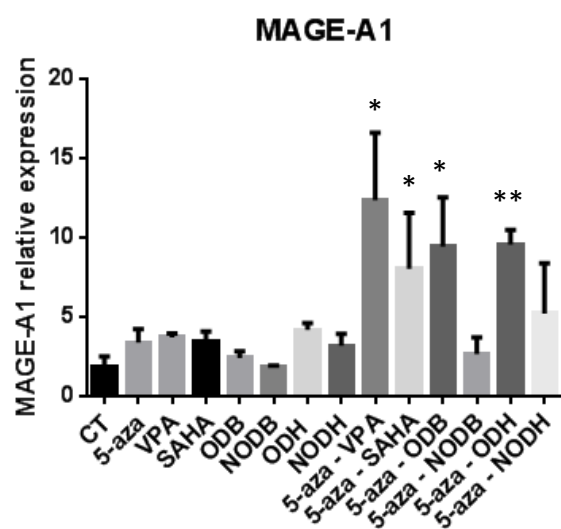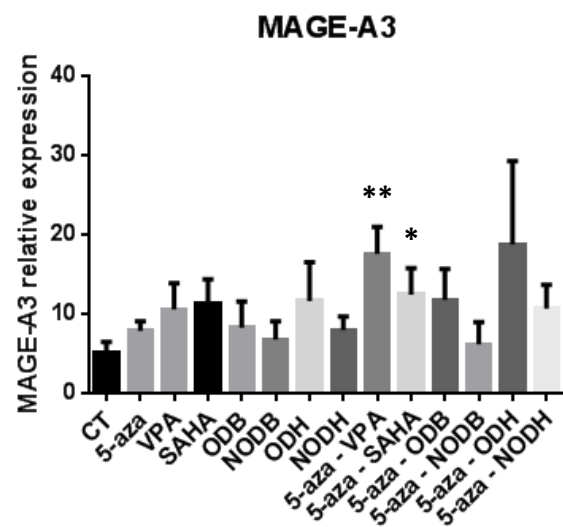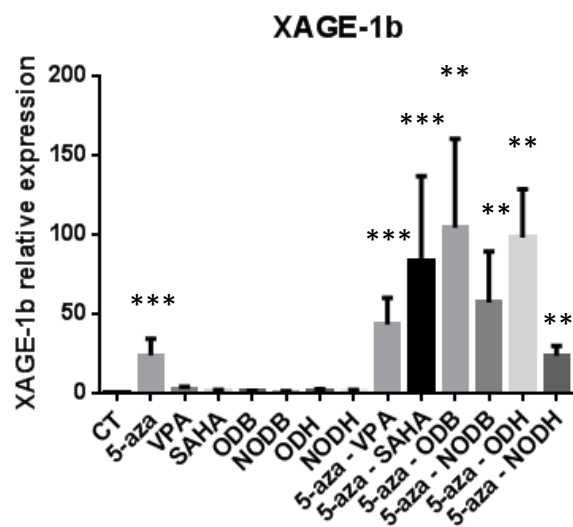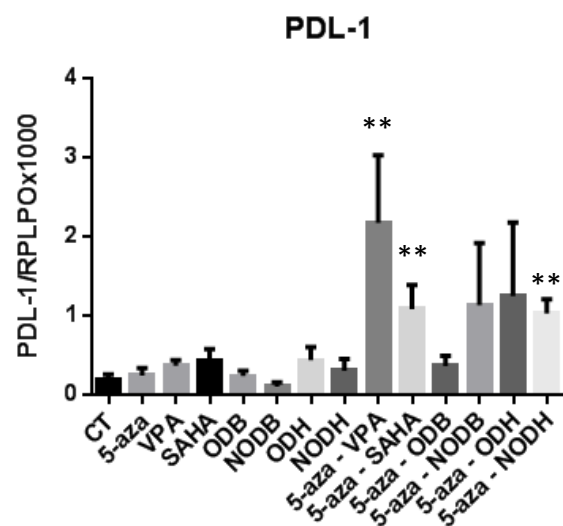

Supplement: Supplementary file 6 — Figure S3. HDACi increase decitabine-induced CTA expression in Meso96 cells. Meso96 cells were treated with: VPA 5 mM, SAHA 2.5 μM, ODB 7.5 μM, NODB 2.5 μM, ODH 2.5 μM, and NODH 25 nM (48 h) in combination or not with decitabine (5-aza) 500 nM (72 h pretreatment). NY-ESO-1, MAGE-A1, MAGE-A3, XAGE-1b and PD-L1 mRNA were measured using real time PCR. (PDF 199 kb) [file 13148_2018_517_MOESM6_ESM.pdf]

Fig. S4

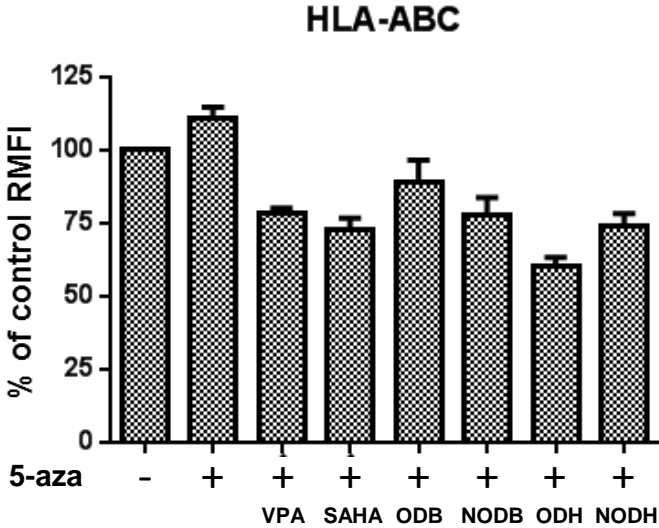

Supplement: Supplementary file 7 — Figure S4. Expression of HLA ABC in Meso96 treated with decitabine/HDACi combinations. Meso96 were treated with: VPA 5 mM, SAHA 2.5 μM, ODB 7.5 μM, NODB 2.5 μM, ODH 2.5 μM, and NODH 25 nM (48 h) in combination or not with decitabine (5-aza) 500 nM (72 h pretreatment). Then, HLA ABC expression was measured using flow cytometry. Results are expressed as the means ± S.E.M of three independent experiments. (PDF 91 kb) [file 13148_2018_517_MOESM7_ESM.pdf]

Fig. S5

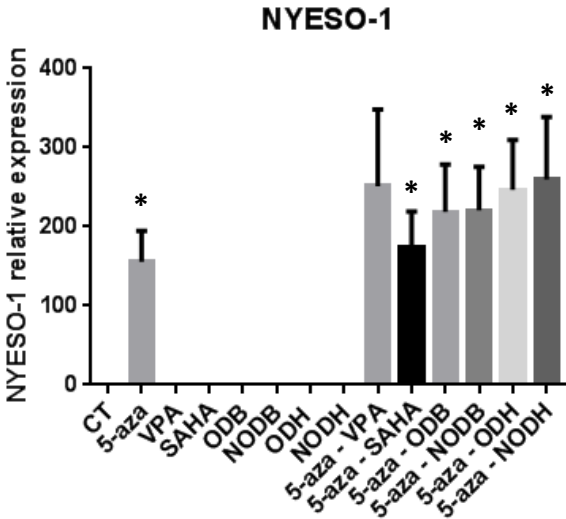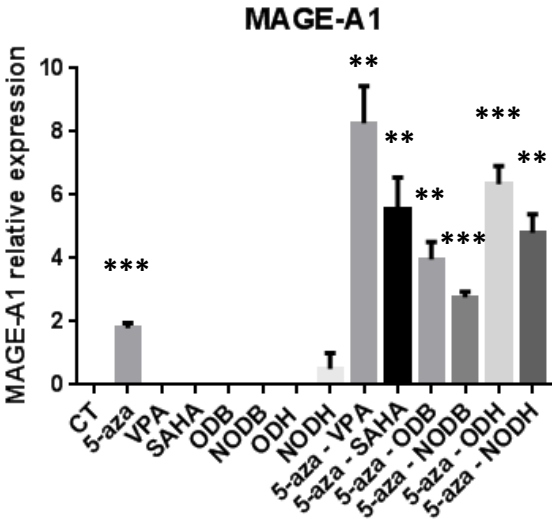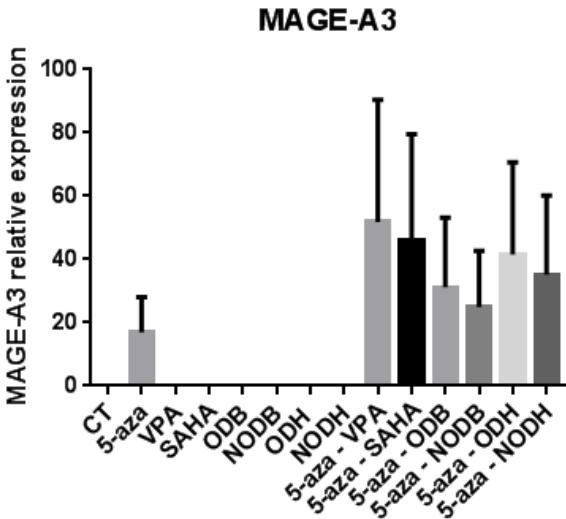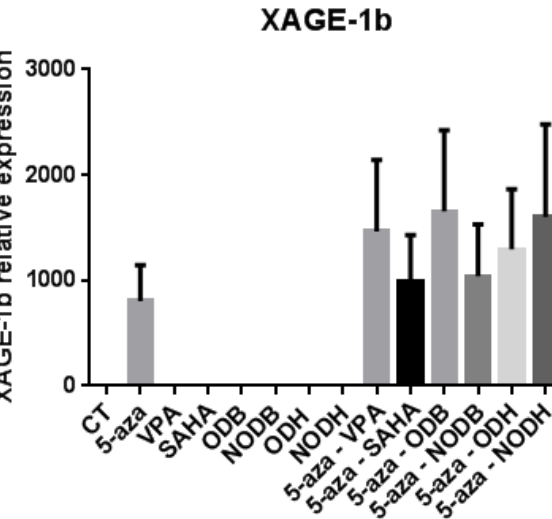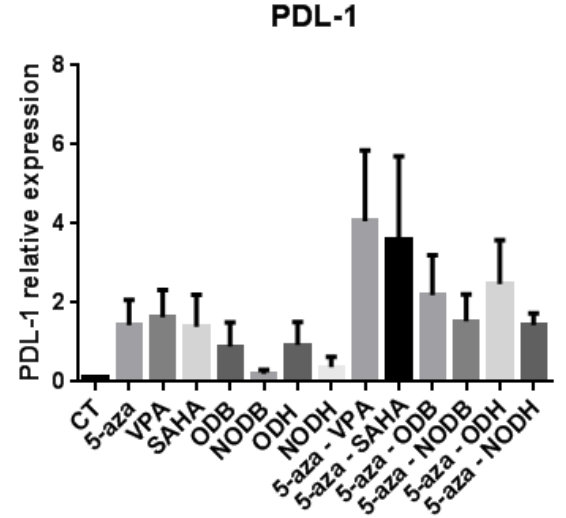

Supplement: Supplementary file 8 — Figure S5. HDACi increase decitabine-induced CTA expression in Meso34 cells. Meso34 cells were treated with: VPA 5 mM, SAHA 2.5 μM, ODB 7.5 μM, NODB 2.5 μM, ODH 2.5 μM, and NODH 25 nM (48 h) in combination or not with decitabine (5-aza) 500 nM (72 h pretreatment). NY-ESO-1, MAGE-A1, MAGE-A3, XAGE-1b and PD-L1 mRNA were measured using real time PCR. (PDF 197 kb) [file 13148_2018_517_MOESM8_ESM.pdf]

Fig. S6

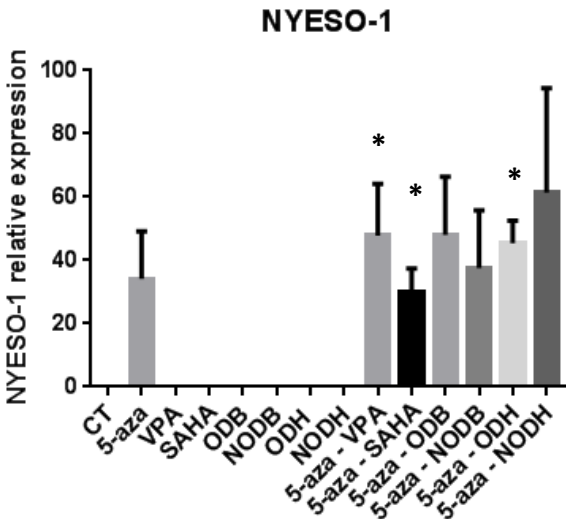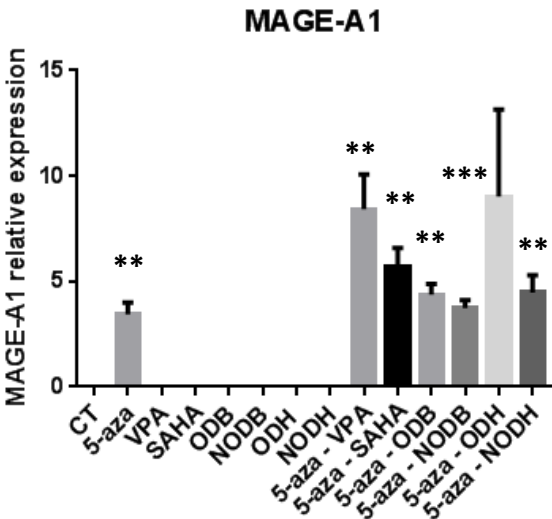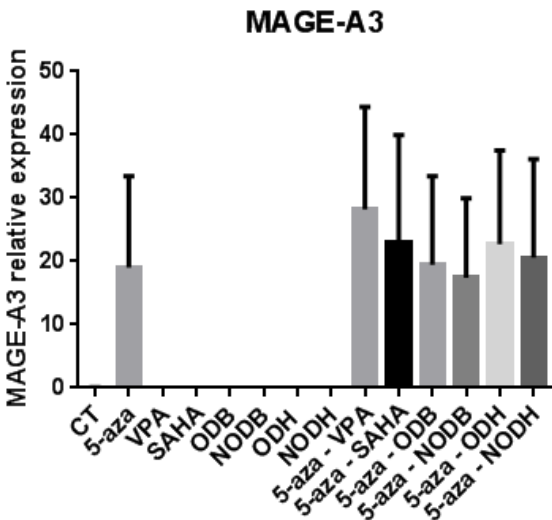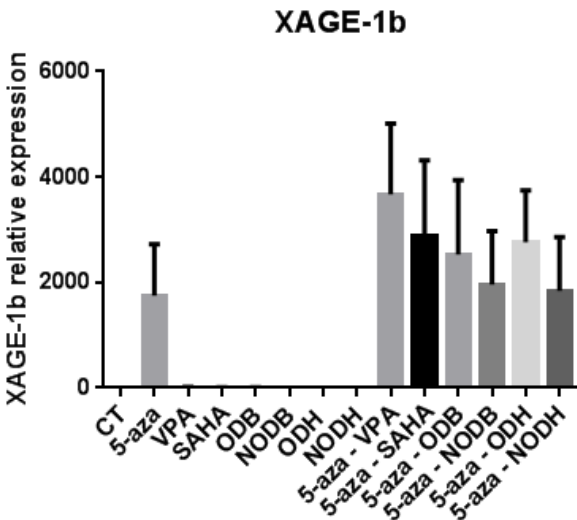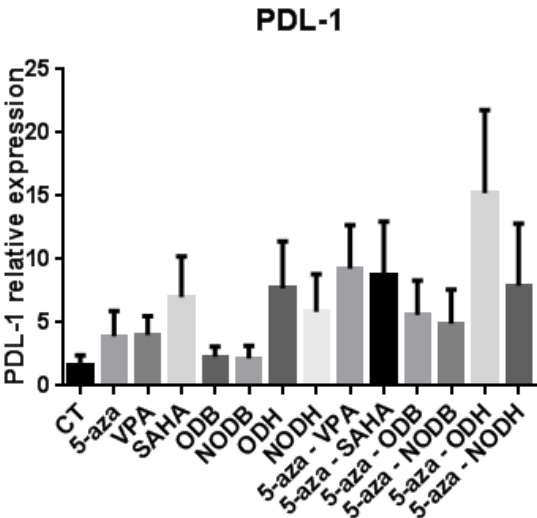

Supplement: Supplementary file 9 — Figure S6. HDACi increase decitabine-induced CTA expression in Meso45 cells. Meso45 cells were treated with: VPA 5 mM, SAHA 2.5 μM, ODB 7.5 μM, NODB 2.5 μM, ODH 2.5 μM, and NODH 25 nM (48 h) in combination or not with decitabine (5-aza) 500 nM (72 h pretreatment). NY-ESO-1, MAGE-A1, MAGE-A3, XAGE-1b and PD-L1 mRNA were measured using real time PCR. (PDF 197 kb) [file 13148_2018_517_MOESM9_ESM.pdf]

Fig. S7

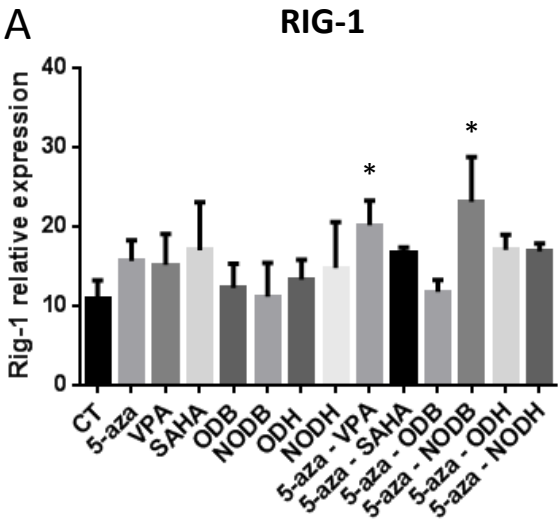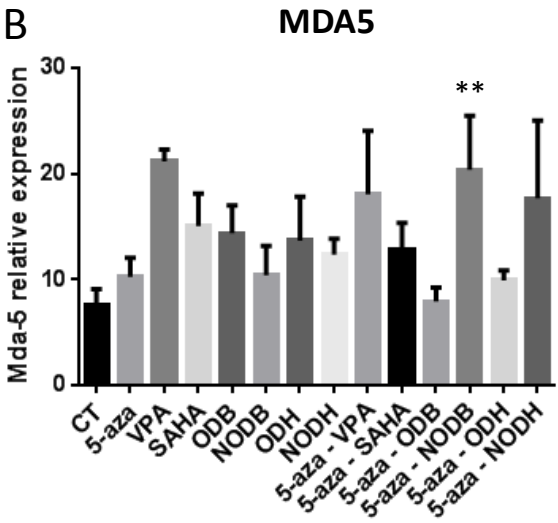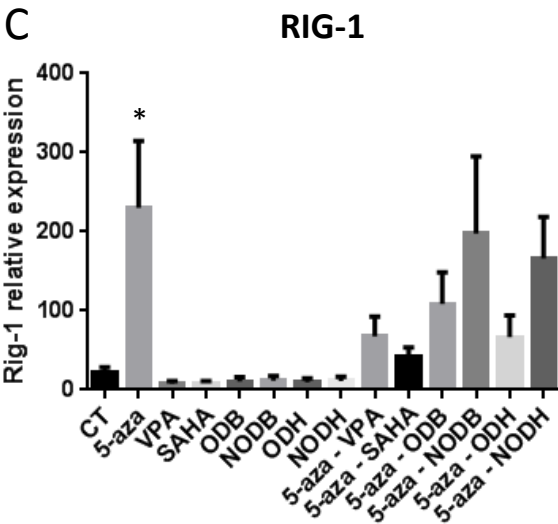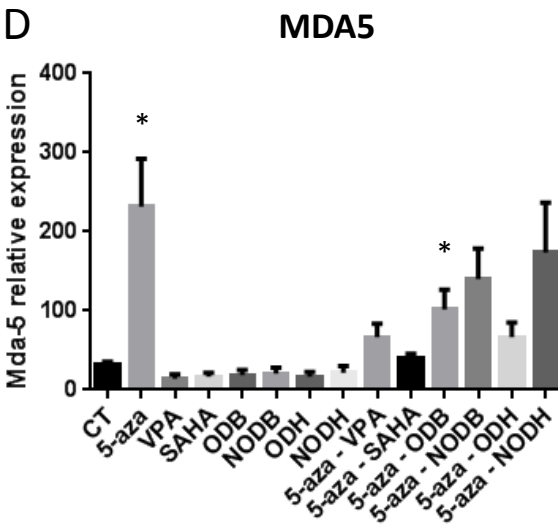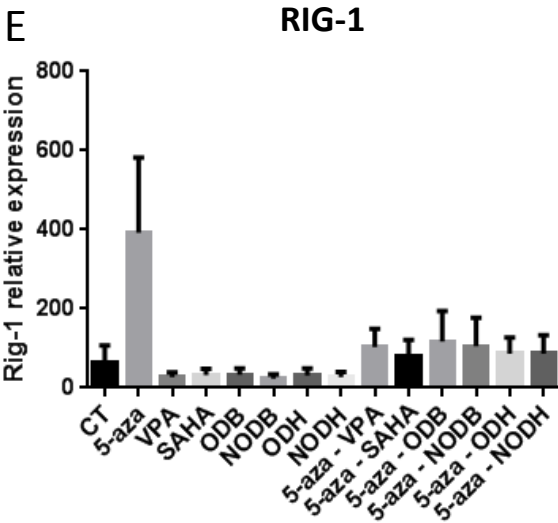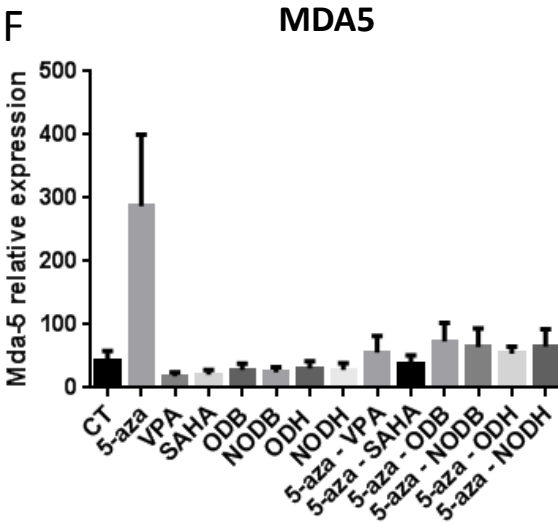

Supplement: Supplementary file 10 — Figure S7. HDACi modulate decitabine-induced RIG-1 and MDA5 expression in MPM cells. Meso34 (top), Meso45 (middle) and Meso96 (down) cells were treated with: VPA 5 mM, SAHA 2.5 μM, ODB 7.5 μM, NODB 2.5 μM, ODH 2.5 μM, and NODH 25 nM (48 h) in combination or not with decitabine (5-aza) 500 nM (72 h pretreatment). RIG-1 (left) and MDA-5 (right) mRNA expression were measured using real time PCR. *p < 0.05, **p < 0.01 and ***p < 0.001. (PDF 209 kb) [file 13148_2018_517_MOESM10_ESM.pdf]

Fig. S8

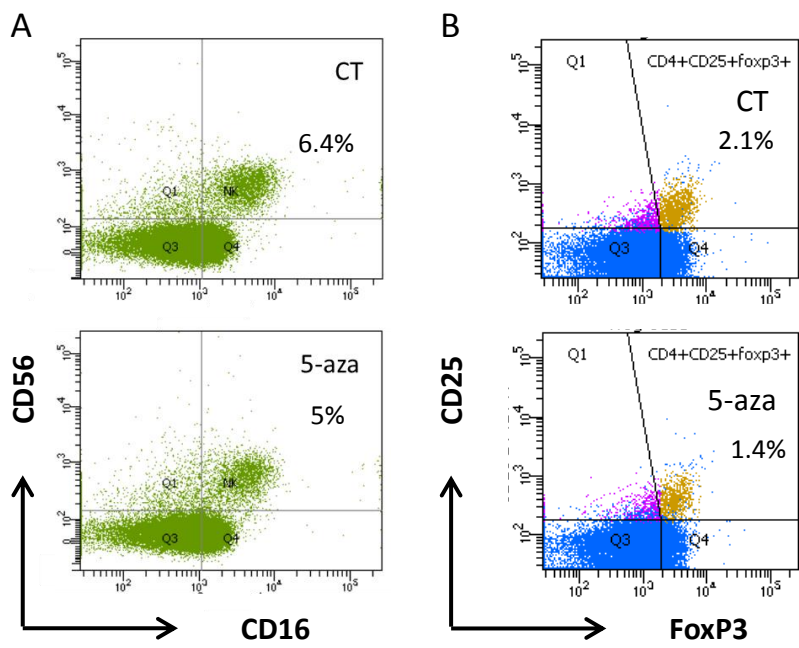

Supplement: Supplementary file 11 — Figure S8. Effect of decitabine on Treg and NK cells. Lymphocytes obtained by elutriation were treated with decitabine (5-aza) 500 nM (72 h). Figure are examples of results obtained on A) natural killer cells (NK) and B) regulatory T cells (Treg) using flow cytometry. (PDF 281 kb) [file 13148_2018_517_MOESM11_ESM.pdf]

Fig. S9

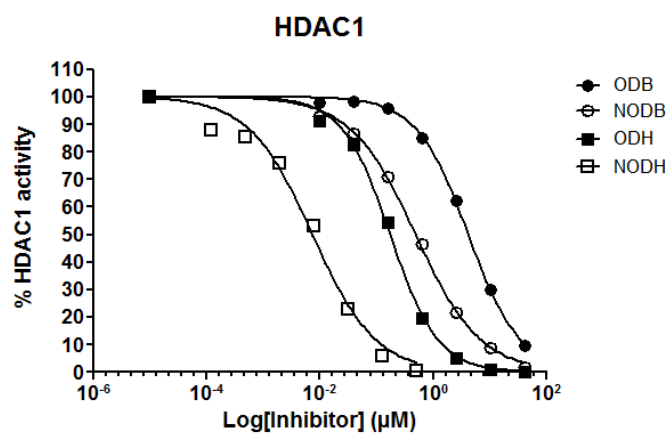

Supplement: Supplementary file 12 — Figure S9. Determination of HDAC1 inhibition properties of ODB, NODB, ODH and NODH. Recombinant HDAC1 activity in the presence of increasing doses of ODB, NODB, ODH and NODH were measured using Fluor de Lys® Drug Discovery Assays (Enzo Life Sciences). (PDF 89 kb) [file 13148_2018_517_MOESM12_ESM.pdf]
